# Parent and Clinician Views of Managing Children with Symptoms of a Lower Respiratory Tract Infection and Their Influence upon Decisions to Take Part in a Placebo-Controlled Randomised Control Trial

**DOI:** 10.3390/antibiotics10040356

**Published:** 2021-03-28

**Authors:** Catherine J. Woods, Zoe Morrice, Nick A. Francis, Paul Little, Theo Verheij, Geraldine M. Leydon

**Affiliations:** 1 Primary Care Research Centre, School of Primary Care, Population Sciences and Medical Education, University of Southampton, Aldermoor Close, Southampton SO16 5ST, UK; Zfm1e15@gmail.com (Z.M.); Nick.Francis@soton.ac.uk (N.A.F.); p.little@soton.ac.uk (P.L.); g.m.leydon@soton.ac.uk (G.M.L.); 2 Julius Center for Health Sciences and Primary Care, University Medical Center Utrecht, 3584 CG Utrecht, The Netherlands; T.J.M.Verheij@umcutrecht.nl

**Keywords:** primary care, respiratory tract infections, children, antibiotic prescribing, qualitative

## Abstract

Children presenting with uncomplicated lower respiratory tract infections (LRTIs) commonly receive antibiotics despite public campaigns on antibiotic resistance. Qualitative interview studies were nested in a placebo-controlled trial of amoxicillin for LRTI in children. Thirty semi-structured telephone interviews were conducted with sixteen parents and fourteen clinicians to explore views of management and decisions to participate in the trial. All interviews were audio-recorded, transcribed and analysed using thematic analysis. Parents found it difficult to interpret symptoms and signs, and commonly used the type of cough (based on sound) to judge severity, highlighting the importance of better information to support parents. Provision of a clinical examination and reassurance regarding illness severity were key motivations for consulting. Many parents now acknowledge that antibiotics should only be used when ‘necessary’, and clinicians reported noticing a shift in parent attitudes with less demand for antibiotics and greater satisfaction with clinical assessment, reassurance and advice. Decisions to take part in the trial were influenced by the perceived risks associated with allocation to a placebo, and concerns about unnecessary use of antibiotics. Clear communication about self-management and safety-netting were identified as important when implementing ‘no antibiotic’ prescribing strategies to reassure parents and to support prescribing decisions.

## 1. Introduction

A large proportion of consultations in primary care involve parents consulting with children with symptoms of a respiratory tract infection (RTI) and antibiotics are frequently prescribed [1]. Overuse of antibiotics is harmful, both for the individual and society, in terms of the side effects associated with taking antibiotics, the harms associated with medicalising minor illnesses, and increased antimicrobial resistance (AMR) [2,3,4,5,6]. Evidence from clinical trials (primarily involving adults) has pointed to the limited efficacy of using antibiotics to treat the symptoms arising from a RTI [3,7] and current clinical guidelines recommend that a ‘no antibiotic’ prescribing strategy should be agreed for most patients, unless the patient is systemically unwell or at high risk of developing complications [5,8].

It is important to understand parent and clinician perceptions about the reasons for consulting in primary care and use of antibiotics for RTI in children as this can help improve healthcare services and appropriate use of antibiotics. Previous qualitative studies have found that parents consult primary care services for children with symptoms of an RTI due to perceptions of illness severity [9,10,11], uncertainty about how to assess illness severity at home [10,12], and with desires for a clinical examination and reassurance from a trusted heath professional [10,13,14,15]. Clinician decision-making to prescribe antibiotics is influenced by perceived clinical need [14,16,17], but also by clinical uncertainty about future deterioration and fears of missing something more serious in a child [11,14,18,19,20].

We conducted a placebo-controlled randomised trial of antibiotics for lower respiratory tract/chest infection (LRTI) in children which has found that amoxicillin for uncomplicated chest infections in children is unlikely to be clinically effective [21]. We nested two qualitative studies, with parents and clinicians, within the trial to explore their views of the trial and its procedures, as well as their experiences of managing a child with symptoms of LRTI, antibiotic use and the factors that might influence antibiotic prescribing. While previous studies have explored clinician and parent views of consulting and antibiotic use, it is important to conduct further qualitative research to explore if perceptions have changed over time due to ongoing high-level campaigns about the dangers of antibiotic resistance and related antibiotic stewardship programmes, and if further support for parents and/or clinicians is needed reduce unnecessary antibiotic prescribing for children with symptoms of an LRTI.

## 2. Materials and Methods

Two nested qualitative studies using semi-structured telephone interviews were carried out by CW with parents and clinicians who participated in ARTIC-PC (Antibiotics for lower Respiratory Tract Infection in Children presenting in Primary Care). Children (between six months and twelve years) presenting to primary care with symptoms of an acute, non-complicated LRTI were eligible to participate. Parents were encouraged to enrol eligible children in the randomised controlled trial of amoxicillin versus placebo, but children not eligible for the trial, or whose parent did not consent for them to take part in the trial, could be enrolled in an observational study with matching outcomes (see Little et al. [21] for a full description of the trial and its results).

All participating parents and clinicians were eligible to take part in the qualitative studies. Parents who had given written informed consent to take part in an interview at the time of recruitment to ARTIC-PC were invited to participate in a telephone interview. The participants were purposefully sampled by whether they took part in the trial or the observational study. A semi-structured topic guide explored parental reasons for consulting and perspectives about antibiotics use, as well as their views about the trial and its procedures and reasons for participation or non-participation in the main trial (see Supplementary Materials). Key topics identified from reviewing relevant literature influenced the initial questions used within this guide, which were then further refined based on discussions in team meetings. The guide was pilot tested with two parents. Parent interviews took place between November 2016–July 2017.

Clinicians participating in ARTIC-PC were also invited to take part in a semi-structured telephone interview, to explore their views of the trial and recruitment, as well as their experiences of managing and treating children with symptoms of an LRTI in primary care. The topic guide for this study was similarly influenced by reviewing relevant literature and refined in team meetings (see Supplementary Materials). The interviews took place between August 2017 to February 2018.

All interviews were audio-recorded, transcribed verbatim and analysed thematically [22]. As the interviews were pragmatic in design and focus (i.e., to elicit views of participating in ARTIC-PC), the analysis aimed to develop themes that reflected the semantic content of participants reported experiences [23]. The six-stage analytic process described by Braun and Clarke was used to inductively code the data line-by-line and develop themes [22]. CW first coded both sets of the interviews about participant views and experiences of trial procedures and participation, to ensure key issues were reported to the study team and the recruitment process could be modified in a timely manner. The remaining aspects of the parent interviews were individually coded by CW and a psychology student (ZM) on a 6-week summer placement in the Department of Primary Care at the University of Southampton. The codes were compared and refined in meetings with CW, ZM and the qualitative work package lead GL. CW coded the remaining clinician interviews and developed the final thematic framework that reflected both datasets. Analyses were facilitated by Nvivo (v11) software.

## 3. Results

A total of 30 participants were interviewed: 16 parents (15 mothers and 1 father) and 14 clinicians (9 GPs, 4 nurse practitioners and 1 research nurse). The parents all consulted with children aged 6 years or under (the youngest child was 9 months). Seven of the parents interviewed took part in the trial and 9 in the observational study. Parent interviews lasted between 20–50 min (average 33 min) and clinician interviews lasted between 25–50 min (average 35 min).

We identified seven themes which reflected the entire dataset. These themes related to parent and clinician views of the trial, its procedures and participation, as well as their experiences of managing a child with symptoms of an LRTI (see Table S1, Supplementary Materials). This paper will summarise key intersecting topics relating to views and experiences of managing a child with symptoms of an LRTI, antibiotic use, and how these views influenced participation in the trial (Table 1).

### 3.1. Parental Reasons for Seeking Consultation and Their Expectations

#### 3.1.1. Symptom Severity and Cause

The perceived severity of the cough, duration of the illness and persistence despite attempts at self-care were key factors that fed into the parents’ decision to consult. Most of the parents had tried to self-manage the child’s illness at home prior to consulting a clinician, most commonly with paracetamol preparations. Cough was a key symptom causing parental concern, and in particular, it was the sound of the cough that seemed to generate concern, with coughs described as ‘rattily’, ‘hacking’, ‘barking’, and ‘wheezy’ causing particular concern, and whether it appeared in combination with other symptoms such as a fever.

#### 3.1.2. Difficulty in Interpreting Symptoms and Previous Experiences of LRTI

Many parents described difficulties in interpreting their child’s signs and symptoms, and in particular being able to differentiate between mild symptoms that might be part of a common minor illness and symptoms that could be indicative of a more serious illness. Several parents reported that previous experience of respiratory illness in their child (or a sibling), which included milder chest infections through to a more serious problems such as pneumonia requiring hospitalisation, influenced how they interpreted their child’s symptoms in the current episode.

#### 3.1.3. Expectations of the Consultation

The main reason the parents made an appointment was to receive a clinical assessment. Parents discussed feeling reassured by clinical examinations as a clinician could listen to the child’s chest and ‘determine the cause and severity of the illness’. A few of the parents said they wanted a prescribed medical treatment which did include antibiotics but also inhalers for an existing respiratory problem. For many parents, the clinical assessment and a diagnosis seemed more important than receiving specific treatments.

#### 3.1.4. Perspectives about Antibiotics and When They Should Be Used

Many of the parents acknowledged that antibiotics should only be used when needed and associated this with having an infection. Some parents offered their understandings about how they could differentiate between a viral and bacterial infection which included explanations such as that an infection will not clear up by itself, that the symptoms are more severe and that they are associated with temperatures or the child acting out of character. Many parents thought that antibiotics were effective and generally worked straight away or within a few days if prescribed correctly.

### 3.2. Clinician Views and Experiences of Managing Children with Symptoms of an LRTI

#### 3.2.1. Decision to Prescribe Antibiotics Is Based on Clinical Indicators and Risk Factors

The clinicians emphasised that they took several factors into account when deciding whether to prescribe antibiotics—the clinical features that the child was presenting with were important, but also the duration of the illness, attempts at self-care, and the patient’s past medical history including risk factors (one clinician described this as ‘the whole picture’).

#### 3.2.2. Parent Expectations and the Importance of Communication

Many clinicians recognised that parents want a thorough clinical examination, reassurance and advice primarily, and some said that they had noticed a shift in parental attitudes with less demand for antibiotics. The clinicians emphasised the importance of communication when explaining the signs (or absence) of an infection during a clinical assessment and providing information about the usual natural history of the illness. Both of which were said to reassure parents and help the clinician elicit (and possibly shape) expectations, and promote acceptance of the treatment outcome.

#### 3.2.3. Prescribing Alternatives to Immediate Antibiotics

The clinicians said that they regularly offered self-care advice when not prescribing immediate antibiotics and sometimes delayed antibiotic prescriptions. Those that had offered delayed prescriptions indicated that they thought that parents were generally accepting of these. Clinicians emphasised the importance of providing safety-netting information when not prescribing antibiotics, to ensure the parent knew what they should do if signs of deterioration occurred after leaving the consultation.

### 3.3. Views of Antibiotic Use and How They Influenced Decisions to Take Part in ARTIC-PC

A few of the parents discussed the dilemma of wanting to do the best by their child and also not wanting them to take antibiotics unnecessarily. Being asked to take part in the trial was considered a positive option in part because it removed the decision-making burden from the parent in terms of what the optimal management plan should be. The parents who took part in the observational study focused more on the risks associated with taking a placebo which was either related to the concept of a placebo itself (e.g., untested medication) or the lack of immediate antibiotics.

The clinicians similarly reported that parental decisions were influenced by the risks associated with taking a chance on a placebo versus immediate antibiotics, and also concerns about their child taking antibiotics unnecessarily. Clinician decisions about whether to enrol the child into the main trial or observational study were also shaped by perceptions of clinical need (or not) for immediate antibiotics.

## 4. Discussion

The main findings of this qualitative study were that parental assessment of the type of cough (assessed primarily by type of sound), the difficulty of interpreting symptoms and signs, and previous experience of RTIs in children, were key factors driving concern about RTIs and the need to attend for clinical assessment and reassurance. Parents demonstrated awareness that antibiotics should only be prescribed when ‘necessary’, but had some misconceptions about identifying bacterial infections and the role of antibiotics. Clinicians reported a general decline in parent expectations and pressure for antibiotics over time, that parents were generally satisfied with a clinical examination and reassurance, and that they used a holistic approach when deciding whether to prescribe antibiotics. Clinicians emphasised the importance of ‘clear communication’ regarding management and safety-netting, especially when implementing a ‘no-antibiotic’ prescribing strategy and had mostly positive views about the acceptability of delayed antibiotic prescriptions.

### 4.1. Comparisons with Existing Literature

The parents within our study displayed varying levels of confidence in assessing the severity of their child’s illness at home and these concerns were key drivers for consulting in primary care. Parental assessments of symptom severity were influenced by the sound of the cough and whether the symptoms had persisted at attempts at self-care [10,13], but these are not predictors of serious illness. A chest examination is also unlikely to detect serious illness in a child who does not have other features associated with a serious illness, and parents could conduct several useful assessments of their child at home (which they may not be aware of, for example, breathing rate, temperature, chest indrawing). The perceived a need for a clinical assessment from a trusted health professional for reassurance that the illness was not indicative of something more serious has been reported in other qualitative studies that have examined parental reasons for consulting in primary with a child with symptoms of an RTI [10,11,13,14,15]. The current study indicates that there are particular areas where better information and support for parents could enable them to assess illness severity at home.

Previous studies have found that fear of future deterioration in a child is a key driver for antibiotic prescribing [11,14,16,20], however the clinicians within this study displayed confidence in their decisions not to prescribe antibiotics. They emphasised the importance of ‘clear’ communication at the time of the consultation, especially around safety netting advice. The provision of safety-netting information is recommended in clinical guidelines [8], but there is a lack of evidence about what information is currently provided in consultations involving sick children. A previous qualitative study found that parents viewed the safety-netting advice provided by their GP at the time of the consultation as unclear and too vague to be useful [24]; and a recent study utilising observational methods similarly found that safety-netting advice in adult consultations was unspecific or not always provided [25]. A study by Francis and colleagues [26] found that written information discussed at the time of the consultation was highly regarded by both parents and GPs and effective at reducing antibiotic prescribing, although it was not always implemented as intended by clinicians. The UK National Institute for Health and Care Excellence (NICE) [27] have produced guidance, including a traffic light system, for assessing serious signs and symptoms in febrile children. However, there is a lack of information on the signs and symptoms that are useful for parents to assess, and how parents can best be supported to assess severe illness in their children. Further research on the optimum delivery of safety-netting advice when implementing ‘no antibiotic’ prescribing strategies may thus be warranted.

The findings from this qualitative process evaluation indicate that parents are willing to accept alternatives to immediate antibiotics and clinicians are confident in their decisions to prescribe alternatives, which is positive based on the results of the overall trial [21], but parental concerns about symptom severity suggest that parents might need to better information and support to manage their child’s illness at home. A systematic review [28] about interventions to modify parent consulting behaviours found that interventions that include specific information about the child’s presenting symptom(s) are more likely to be effective and reduce consultation rates for RTIs than interventions that include generic messages about antibiotic overuse or resistance (see also [14,29,30,31]. There is currently conflicting evidence about whether this information would be most effective if delivered during the consultation or prior to the illness episode [28,30]. Future research is needed to better understand whether parents would be happy to take more responsibility for assessing their child at home, the format parents would prefer this information, and when it would be most effective to facilitate home management.

### 4.2. Strengths and Limitations

This process evaluation provides insights into how perceptions of illness severity and management can affect parent and clinician decision-making regarding appropriate treatment for a child with an LRTI and participation in placebo-controlled trial. There was agreement across the two datasets regarding changing preferences for antibiotics which provides stronger evidence than if the study was based on parents or clinicians alone. The main limitations of our study are that the results are based on a small sample size of each type of participant. The parents interviewed agreed to take part in the trial or the observational study, and so we did not explore the views of parents who declined to take part in ARTIC-PC and how they compare to the parents we recruited. It should also be noted that parents might have expressed less concern about their child receiving antibiotics because they were enrolled in a research study and would have access to care if their child deteriorated. This may differ to acceptance of no antibiotics in usual consultations, although the clinicians in this study did report a decline in parent expectations for antibiotics.

## 5. Conclusions

This study found that parents primarily consult primary care services due to particular concerns about how to interpret cough and illness severity, and a desire for reassurance. Many parents are happy to accept alternatives to immediate antibiotics, including self-care advice and delayed prescriptions. The clinicians we interviewed displayed confidence in their ability to decide when to not prescribe antibiotics, but other research has shown that fear of deterioration and concerns of safety can lead clinicians to unnecessary prescribing for children. To reduce consultations for children with an RTI, better information and support might be needed for parents that will enable them to assess illness severity at home. Additionally, further research might also explore the optimum delivery of safety-netting information as this was identified as crucial for implementing ‘no antibiotic’ prescribing strategies.

## Figures and Tables

**Table 1:** Summary of key topics.

Themes	Subthemes	Illustrative Quote(s)
**3.1. Parental reasons for seeking consultation and their expectations**	**3.1.1. Symptom severity and cause**	*“…it was this sort of, it was like that rattly, kind of quite tight cough that you could tell was sort of hurting her, and then when she woke up on the morning after the doctor’s appointment she was able to [eat]. She was able to tell me that her chest was hurting, so, well she didn’t actually say her chest but she pointed, so, er, I knew, I knew that actually, that’s probably what it was”* (Parent 13) *“…so he’d had something for about a week or so kind of ongoing cough that hadn’t really seemed to get any better… He was going off his food, which is quite a big change for him because he usually eats an awful lot, and things didn’t seem to be getting any better and he was starting to sound more congested and raspy and a little bit wheezy as well, so we thought right, let’s take him down and get somebody to check him”* (Parent 2)
	**3.1.2. Difficulty in interpreting symptoms and previous experiences of LRTI**	*“INT: Okay. So what made you book an emergency appointment?* *PAR: Because, in the past when she had chest infections, if I didn’t sort of jump on it quite quickly, it then took about two weeks to get rid of it. So, if, you know, the faster I sort of solved it, the easy, you know, the sort of less extreme it was for her. Erm, but before, when we kind of left it a couple of days it had then taken a while and it was quite hard to get rid of it”* (Parent 13)
	**3.1.3. Expectations of the consultation**	*“I just wanted them to check her chest really and make sure it was definitely clear…”* (Parent 4) *“I wouldn’t go to the doctor and say, ‘I want antibiotics’ but I know that can happen. I would want, in their medical opinion, what is best to treat my child”* (Parent 5)
	**3.1.4. Perspectives about antibiotics and when they should be used**	*“Yes, well antibiotics are if it’s a bacterial infection and one which is having quite significant symptoms, or signs that the body is not fighting it off or that it’s likely to develop into something else, then yes”* (Parent 3)
**3.2. Clinician views and experiences of managing children with symptoms of an LRTI**	**3.2.1. Decision to prescribe antibiotics is based on clinical indicators and risk factors**	*“I think, if a child had very definite unilateral symptoms, so, crackles on their chest, I’d probably be more likely to give antibiotics. Occasionally, if they had a concurrent illness, or respiratory problem, so, an asthmatic, or something like that, I might be more prone to give antibiotics as a caution.”* (GP5)
	**3.2.2. Parent expectations & the importance of communication**	*“INT: If you’re not going to prescribe antibiotics what kind of things do you suggest to parents?* *PAR: We do safety netting which involves talking about the natural history of the disease, telling people what to look out for and when I want to see them again. So if it’s a chest infection, since we’re talking about ARTIC, I’d talk about increasing breathlessness, temperature that’s hard to control, lethargy, off food, anything like that we have another look at them. I say, ‘I can’t hear anything right now, but things might have changed and therefore I’ll review the child because they get ill quickly and get better quickly so there be new signs that I can’t see now, but will have developed in one or two days’ time.’ So it’s just telling them what to look out for so that we don’t miss anything.* *INT: Are parents okay with this usually?* *PAR: Yes”* (GP6) *“INT: How would you go about managing patient expectations in your own consultation?* *PAR: I think you’ve got to find out what they want really and I think allowing the patient to speak, but also exploring it directly, so asking about their expectations is good communication really…so to find out whether they want antibiotics or not is…a powerful piece of information. Interestingly I have found myself forgetting to do that sometimes recently and explaining to patients antibiotics are unlikely to work for their viral bronchitis or whatever infection, they’ve actually said to me they weren’t expecting antibiotics!”* (GP7)
	**3.2.3. Prescribing alternatives to immediate antibiotics**	*“They like it [delayed scripts], because it gives them a little bit of empowerment I think, and allows them to feel a bit more confident of the fact that they don’t have to give antibiotics now and they can wait and see and they often quite like it. That seems to be the rationale! Certainly sometimes if their child had bronchiolitis severely when they were a baby and they’re now four and presenting with a cough, and their child had to go into hospital when they were a baby or whatever, they think back to that time. So actually giving them antibiotics, giving them a plan as to what to do over the next few days if things do deteriorate, they’re very happy and they like that.”* (GP2)
**3.3. Parent views of antibiotic use and how they influenced decisions to take part in ARTIC-PC**	**Parents**	*“… I don’t like to give them anything really, unless I feel that they really need it, even down to Calpol and stuff, unless the fever is really bad. I don’t tend to give them it just because they’ve had a little bit of a bump on the leg or anything like that, so I felt that the trial was a bit of a happy compromise for me, because in a way I didn’t know if he was actually having anything put into him, and so I could maybe convince myself that it was a placebo. Obviously, he would either get better or get worse but, either way, the care he would be given would be almost a bit better than what it would be if he wasn’t part of it, I suppose”* (Parent 9) *“It’s not fair on [name] if she is feeling poorly and I don’t get the medicine to fix her, so I didn’t want to risk it”* (Parent 5)
	**Clinicians**	*“I think that’s been a big sea change, so a couple of parents who I’ve talked to about taking part in the actual study have said, ‘I don’t want my child to have antibiotics unless they really need it’. So I’m happy to do the observational part, but I don’t want them to have antibiotics unless they need it”* (GP2) *“The other concern I have is the fact that most parents, if they’ve got an ill child, especially if they’re young, they do seem to want to have antibiotics, want them to have antibiotics…If they take part in the study, they may get the placebo, they don’t really want to risk that, despite explaining that in previous studies antibiotics have not proven to be that effective in children with chest infections”* (Nurse 4)

## Supplementary Materials

The following are available online at https://www.mdpi.com/article/10.3390/antibiotics10040356/s1, Topic guides; Table S1: Full list of themes identified across the dataset.

## References

[1] Hay A.D., Heron J., Ness A. (2005). The prevalence of symptoms and consultations in pre-school children in the Avon Longitudinal Study of Parents and Children (ALSPAC): A prospective cohort study. Fam. Pract..

[2] Clavenna A., Bonati M. (2009). Adverse drug reactions in childhood: A review of prospective studies and safety alerts. Arch. Dis. Child..

[3] Moore M., Stuart B., Coenen S., Butler C.C., Goossens H., Verheij T.J.M., Little P. (2014). Amoxicillin for acute lower respiratory tract infection in primary care: Subgroup analysis of potential high-risk groups. Br. J. Gen. Pract..

[4] Little P., Gould C., Williamson I., Warner G., Gantley M., Kinmonth A.L. (1997). General practice Reattendance and complications in a randomised trial of prescribing strategies for sore throat: The medicalising effect of prescribing antibiotics. BMJ.

[5] Public Health England (2020). Summary of Antimicrobial Prescribing Guidance: Managing Common Infections PHE Context, References and Rationales for Clinical Commissioning Groups, Commissioning Support Units and Primary Care Providers.

[6] Department of Health (2019). Tackling Antimicrobial Resistance 2019–2024: The UK’s Five-Year National Action Plan.

[7] Smith S.M., Fahey T., Smucny J., Becker L.A. (2017). Cochrane Library Cochrane Database of Systematic Reviews Antibiotics for acute bronchitis (Review). Cochrane Database Syst. Rev..

[8] National Institute for Clinical Excellence (2008). Respiratory Tract Infections (Self-Limiting): Prescribing Antibiotics.

[9] Wyke S., Russell I., Hewison J., Russell I.T. (1990). Cervical screening View project Respiratory illness in children: What makes parents decide to consult?. Br. J. Gen. Pract..

[10] Ingram J., Cabral C., Hay A.D., Lucas P.J., Horwood J. (2013). Parents’ information needs, self-efficacy and influences on consulting for childhood respiratory tract infections: A qualitative study. BMC Fam. Pract..

[11] Cabral C., Lucas P.J., Ingram J., Hay A.D., Horwood J. (2015). “It’s safer to …” parent consulting and clinician antibiotic prescribing decisions for children with respiratory tract infections: An analysis across four qualitative studies. Soc. Sci. Med..

[12] Francis N., Wood F., Simpson S., Hood K., Butler C.C. (2008). Developing an ‘interactive’ booklet on respiratory tract infections in children for use in primary care consultations. Patient Educ. Couns..

[13] Halls A., Hoff C.V., Little P., Verheij T., Leydon G.M. (2017). Qualitative interview study of parents’ perspectives, concerns and experiences of the management of lower respiratory tract infections in children in primary care. BMJ Open.

[14] Lucas P.J., Cabral C., Hay A.D., Horwood J. (2015). A systematic review of parent and clinician views and perceptions that influence prescribing decisions in relation to acute childhood infections in primary care. Scand. J. Prim. Heal. Care.

[15] Francis N.A., Crocker J.C., Gamper A., Brookes-Howell L., Powell C., Butler C.C. (2010). Missed opportunities for earlier treatment? A qualitative interview study with parents of children admitted to hospital with serious respiratory tract infections. Arch. Dis. Child..

[16] Brookes-Howell L., Hood K., Cooper L., Coenen S., Little P., Verheij T., Godycki-Cwirko M., Melbye H., Krawczyk J., Borras-Santos A. (2012). Clinical influences on antibiotic prescribing decisions for lower respiratory tract infection: A nine country qualitative study of variation in care. BMJ Open.

[17] Little P., Dorward M., Warner G., Stephens K., Senior J., Moore M. (2004). Importance of patient pressure and perceived pressure and perceived medical need for investigations, referral, and prescribing in primary care: Nested observational study. BMJ.

[18] Anderson E.C., Kesten J.M., Lane I., Hay A.D., Moss T., Cabral C. (2019). Primary care clinicians’ views of paediatric respiratory infection surveillance information to inform clinical decision-making: A qualitative study. BMJ Paediatr. Open.

[19] Horwood J., Cabral C., Hay A.D., Ingram J. (2016). Primary care clinician antibiotic prescribing decisions in consultations for children with RTIs: A qualitative interview study. Br. J. Gen. Pract..

[20] Tonkin-Crine S., Yardley L., Little P. (2011). Antibiotic prescribing for acute respiratory tract infections in primary care: A systematic review and meta-ethnography. J. Antimicrob. Chemother..

[21] Little P., Francis N.A., Stuart B., O’Reilly G., Thompson N., Becque T., Hay A.D., Wang K., Sharland M., Harnden A. Antibiotics for lower Respiratory Tract Infection in Children presenting in Primary Care (ARTIC PC): A placebo controlled trial.

[22] Braun V., Clarke V. (2006). Using thematic analysis in psychology. Qual. Red. Psychol..

[23] Braun V., Clarke V. (2019). Reflecting on reflexive thematic analysis. Qual. Res. Sport Exerc. Health.

[24] Cabral C., Ingram J., Hay A.D., Horwood J. (2014). “They just say everything’s a virus”—Parent’s judgment of the credibility of clinician communication in primary care consultations for respiratory tract infections in children: A qualitative study. Patient Educ. Couns..

[25] Edwards P.J., Ridd M.J., Sanderson E., Barnes R.K. (2019). Safety netting in routine primary care consultations: An observational study using video-recorded UK consultations. Br. J. Gen. Pract..

[26] Francis N.a., Phillips R., Wood F., Hood K., Simpson S., Butler C.C. (2013). Parents’ and clinicians’ views of an interactive booklet about respiratory tract infections in children: A qualitative process evaluation of the EQUIP randomised controlled trial. BMC Fam. Pract..

[27] National Institute for Clinical Excellence (2019). Fever in Under 5s: Assessment and Initial Management. https://WwwNiceOrgUk/Guidance/Ng143.

[28] Andrews T., Thompson M., Buckley D.I., Heneghan C., Deyo R., Redmond N.M., Lucas P.J., Blair P.S., Hay A.D. (2012). Interventions to Influence Consulting and Antibiotic Use for Acute Respiratory Tract Infections in Children: A Systematic Review and Meta-Analysis. PLoS ONE.

[29] Van Hecke O., Butler C.C., Wang K., Tonkin-Crine S. (2019). Parents’ perceptions of antibiotic use and antibiotic resistance (PAUSE): A qualitative interview study. J. Antimicrob. Chemother..

[30] Vodicka T.A., Thompson M., Lucas P., Heneghan C., Blair P.S., I Buckley D., Redmond N., Hay A.D. (2013). Reducing antibiotic prescribing for children with respiratory tract infections in primary care: A systematic review. Br. J. Gen. Pract..

[31] Szymczak J.E., Feemster K.A., Zaoutis T.E., Gerber J.S. (2014). Pediatrician Perceptions of an Outpatient Antimicrobial Stewardship Intervention. Infect. Control. Hosp. Epidemiology.

