# Supplementary material for: Parent and Clinician Views of Managing Children with Symptoms of a Lower Respiratory Tract Infection and Their Influence upon Decisions to Take Part in a Placebo-Controlled Randomised Control Trial
Source: Antibiotics (Basel). 2021 Mar 28;10(4):356. doi: 10.3390/antibiotics10040356 (PMC8065881; doi:10.3390/antibiotics10040356)
Supplement: Supplementary file 1 [file antibiotics-10-00356-s001.zip › SupplementaryMaterials_ClinicianTopicGuide.docx]

**ARTIC-PC**


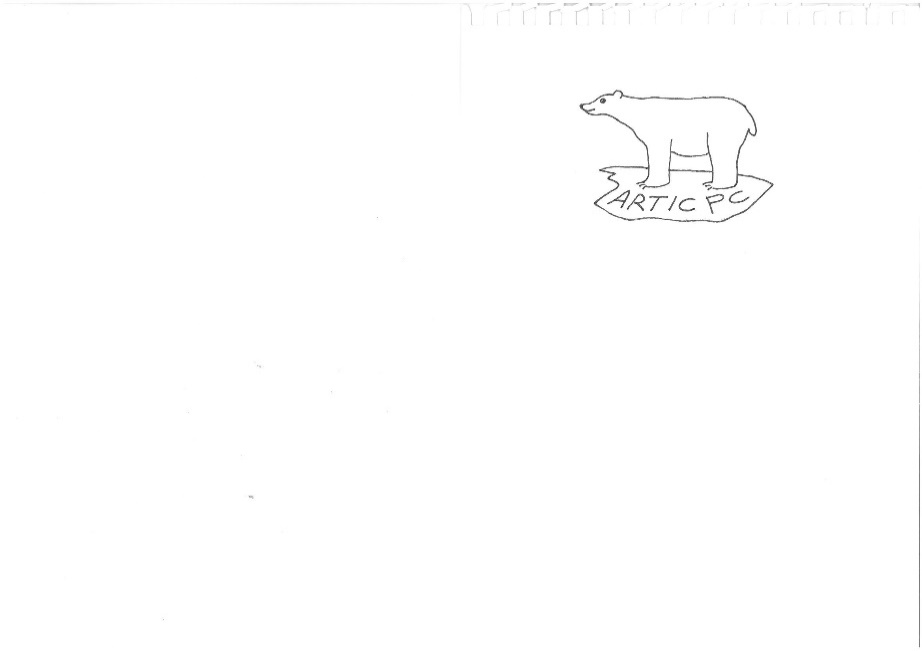
**A**ntibiotics for Lower **R**espiratory **T**ract **I**nfection in **C**hildren Presenting in **P**rimary **C**are

**Nested Qualitative GP Interview Study**

**Researcher:** Dr. Catherine J. Woods

**Main supervisor for the qualitative study:** Prof. Geraldine M. Leydon

**Secondary supervisors:** Prof. Paul Little & Dr. Kim Harman

**Interview Preliminaries**

- Hello could I speak to [**name**]?
- Hi, my name is Catherine and I am a researcher on the ARTIC-PC study.
  - I recently scheduled this interview so we could discuss your involvement within this trial, is now still a good time to chat?
- So firstly, thank you for agreeing to take part in the trial and in the interview – myself and the team really appreciate it and I am looking forward to hearing your views.
- The interview will last between 30 to 40 minutes and we will discuss you views of the trial as well as prescribing antibiotics in primary care.
- I might be quiet during the interview, more so than a usual conversation, but do not let that put you off – I will be listening to your story and possibly jotting things down as we talk.
- Before we begin can I confirm for this interview to be audio-recorded, anonymised, transcribed and used in our analysis?
  - Reason we ask…thorough record of what we discussed.
  - The recording and transcript will be stored securely at all times and all identifiable information will be removed so that nothing can be linked back to you directly.
  - You can change your mind at any point about taking part in the study, even after this interview has been conducted; and you can also decline to answer any of my questions if you do not want to.
- At this point, do you have any questions for me?
- I am now turning the recorder on.

**Participation in the Study**

**Could you start by telling me a bit about your general impressions of the study and why you agreed to take part?**

- How did you find the study materials? (*easy/difficult to read, amount, layout*).
- What was your experience of contact with the study team?
- What was you experience of identifying and recruiting eligible participants to ARTIC?

Prompts:

- - Time to recruit.
  - Parents willing to give time.
  - Identification of suitable children.
  - Difference to usual prescribing practices as includes placebo.
- Were there any aspects of the study that you thought were problematic or did not work well? Could you explain why/why not?
- Were there any aspects of the study that you particularly liked? Could you explain why/why not?

**Views of abx, AMR & placebos**

**As you know the trial was a placebo-controlled trial to explore whether abx are effective at treating children with LRTI, could you tell me about…**

1. Your **approach** to prescribing abx for children with LTRI? (*looking for decision-making, key drivers and how/if these compare to adult patients*).
   1. **Prompt:** Are there key history and examination findings that make you more likely to prescribe abx for children with LTRI? (*e.g. age, possible complications*).
   2. **Prompt:** Have you ever come across cases where abx were not effective in treating children with LRTI? (*what happened*).
   3. **Prompt:** If you decide not to prescribe antibiotics, what are the alternatives you tend to suggest to parents for their children?
   4. **Prompt:** Do you ever feel pressure when deciding whether to prescribe/not prescribe abx? (*how do you feel ‘pressured’, are these pressures coming from within the consultation or are they external constraints?*).
   5. **Prompt:** Do you feel the drivers for prescribing decisions in children are different when compared with prescribing for adults? (*how so?)*
   6. **Prompt:** Overall, are you content with the way you manage LRTI in children and the ways you are supported? (*how improve; barriers to improving*).
2. Can you tell me a bit about your views of **antibiotic resistance**?
   1. **Prompt:** Do you think AMR is a problem within your practice?
   2. **Prompt:** Is it something you have discussed with colleagues?
   3. **Prompt:** Would it be helpful to know more about how your peers tackle the

prescribing challenges within your practice/in practices elsewhere in the UK?

- 1. **Prompt:** How do you see AMR developing in the near future? (*e.g. reduce threat*).

**BRIDGE:** Moving on to the second aspect of the trial…

1. What are your views of **using placebos** in primary care?
   1. **Prompt:** Do you have experience of prescribing placebos in your practice or as part

of a previous study?) (*key influences over beliefs*).

- 1. **Prompt:** Use of placebos in RCTs (*easy for GPs to explain to patients?).*
  2. **Prompt:** How do you perceive patient reactions to the concept of placebo? (*if*

*recruited, any examples from ARCTIC PC?*).

- 1. **Prompt:** Is this a topic you discuss / have discussed with peers?

**BRIDGE:** Thank you for that. Going to move on now to the last part of the interview and ask about your views about the current guidance and training about prescribing antibiotics available to healthcare practitioners.

**Views of current evidence base and training**

1. How do you feel about the **current guidance** to support your prescribing decisions for antibiotics [*You can refer to either children or adult patients*]?
   1. **Prompt:** Is this guidance clear/ realistic? Are there some illnesses/ infections not

clear?

- 1. **Prompt:** Guidance for routine consultations and OOH?
  2. **Prompt:** How do you feel about the current **communication guidance** about how to

discuss treatment decisions with patients?

- - 1. Do you feel you need more training?
    2. Our group are looking at developing a communication intervention to help patients and their GPs discuss symptoms and the necessity or otherwise for antibiotics. This training would involve video-recorded consultations, what do you think about that?
    3. Have you come across this form of training before (e.g. medical school)?
    4. Do you think it would be useful to see real recorded instances of GPs discussing antibiotics with their patients for you and colleagues to view/learn from?
    5. What kind of training format would be useful (f2f, online)?

**Bridge:** Okay well that is the end of the questions I have for you. Is there anything else that you would like to add [*about the trial, prescribing abx*] that we have not covered so far?

**Interview Close**

- Thank you so much for your time today and sharing your story with me – it was lovely to speak to you.
- Thank you for letting me record this interview - I am switching the recorder off now *(just have to find the button…).*
- Are you still happy for us to anonymise this recording and use it for our study?
- Do you have any questions for me?

Thank you so much again for taking part in the study and speaking with me today – I hope you have a [*lovely day, nice weekend, good week*].
