# Supplementary material for: Parent and Clinician Views of Managing Children with Symptoms of a Lower Respiratory Tract Infection and Their Influence upon Decisions to Take Part in a Placebo-Controlled Randomised Control Trial
Source: Antibiotics (Basel). 2021 Mar 28;10(4):356. doi: 10.3390/antibiotics10040356 (PMC8065881; doi:10.3390/antibiotics10040356)
Supplement: Supplementary file 1 [file antibiotics-10-00356-s001.zip › SupplementaryMaterials_full list of themes.docx]

**SUPPLEMENTARY MATERIALS**

Table S1 summarises the seven themes identified from the analysis: 3 within the parent interviews and 4 within the clinician interviews. The themes/subthemes highlighted are the ones we reported in this paper because they were the most important in relation to understanding clinician and parent views of consulting and antibiotic use, and how these shaped parental decisions about whether to take part in the main trial (which involved randomisation), or the observational study.

**Table S1 : Full list of themes identified across the dataset**

| **Themes** | **Subthemes** |
| --- | --- |
| **Parent interviews** | |
| **Theme 1: Reasons for consulting** | - Symptom severity and cause |
|  | - Difficulty in interpreting symptoms and previous experiences of LRTI |
|  | - Expectations of the consultation |
| **Theme 2: Parent perspectives & understandings about antibiotic use & antibiotic resistance** | - Perspectives about antibiotics and when they should be used |
|  | - Understandings of antibiotic resistance |
| **Theme 3: Views of taking part in the trial and its procedures** | - Motivations for taking part in the study |
|  | - Positive aspects of taking part overall |
|  | - The study materials were easy to follow or administer |
|  | - Perspectives and understandings about placebos |
|  | - Parent willingness for their child to have the trial medication |
|  | - Perceptions of illness recovery for children who took part in the RCT |
| **Clinician interviews** |  |
| **Theme 1: Antibiotic prescribing for children with LRTI & other common infections** | - Decision to prescribe antibiotics is based on clinical indicators and risk factors |
|  | - Prescribing alternatives to immediate antibiotics |
|  | - Parent expectations & the importance of communication |
| **Theme 2: Views about antimicrobial resistance** | - Antimicrobial resistance is caused by overprescribing antibiotic |
|  | - Strategies to combat AMR |
| **Theme 3: Positive views of taking part in the trial and its procedures** | - The topic of the trial is important |
|  | - The trial procedures were clear & easy to follow |
|  | - The clinicians felt supported by the study team |
|  | - Many parents were willing to take part |
| **Theme 4: Factors affecting recruitment** | - The recruitment process is too time-consuming |
|  | - Opportunistic recruitment is challenging |
|  | - Children not eligible to participate |
|  | - Parents were unwilling to take part |
|  | - Clinician concerns and phrasing of the trial |
