# Supplementary material for: Parent and Clinician Views of Managing Children with Symptoms of a Lower Respiratory Tract Infection and Their Influence upon Decisions to Take Part in a Placebo-Controlled Randomised Control Trial
Source: Antibiotics (Basel). 2021 Mar 28;10(4):356. doi: 10.3390/antibiotics10040356 (PMC8065881; doi:10.3390/antibiotics10040356)
Supplement: Supplementary file 1 [file antibiotics-10-00356-s001.zip › SupplementaryMaterials_ParentTopicGuide.docx]

**ARTIC-PC**

**A**ntibiotics for Lower **R**espiratory **T**ract **I**nfection in **C**hildren Presenting in **P**rimary **C**are

**Nested Qualitative Parent/Child Interview Study**


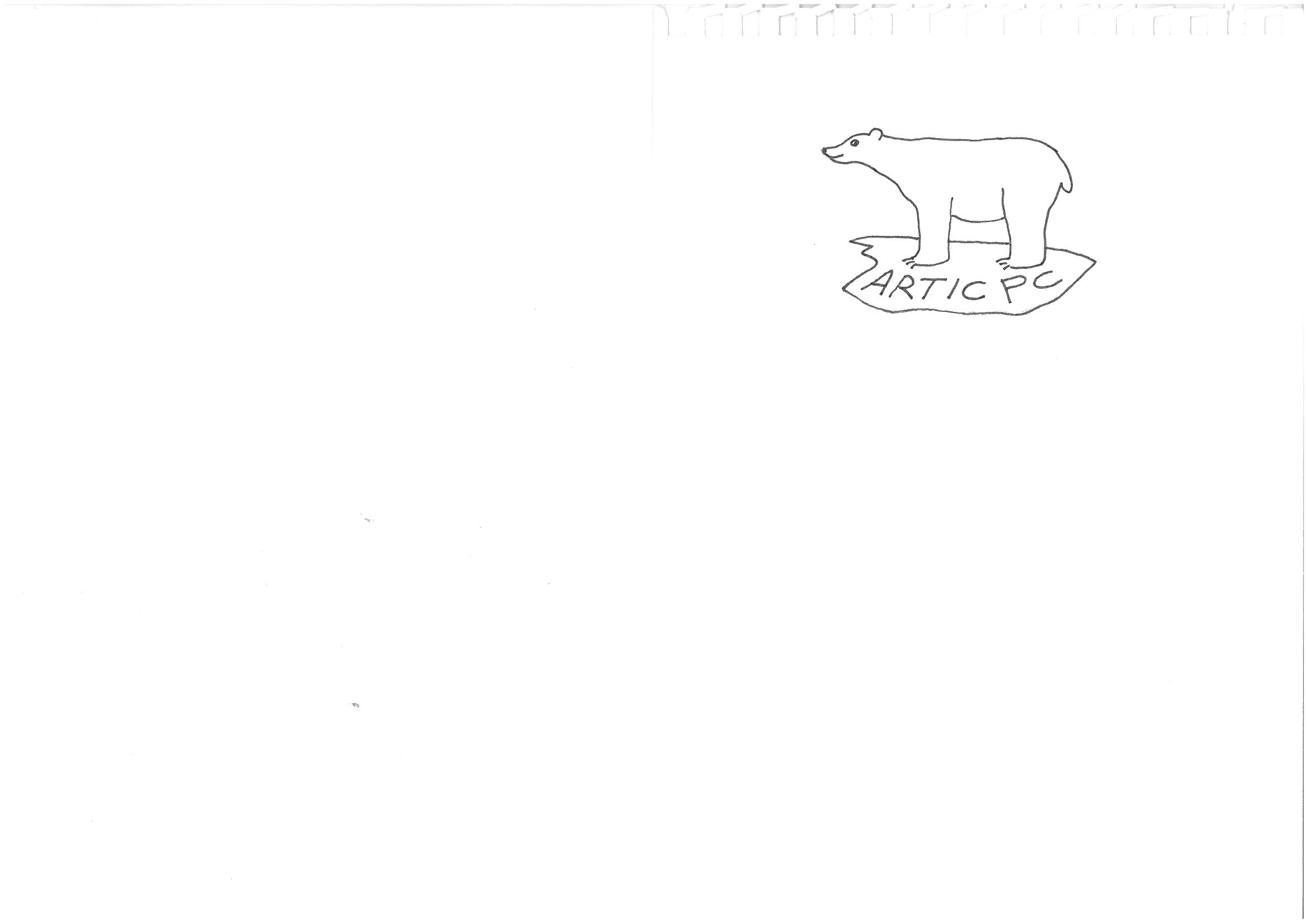


**Researcher:** Dr. Catherine J. Woods

**Main supervisor for the qualitative study:** Dr. Geraldine M. Leydon

**Secondary supervisors:** Prof. Paul Little & Dr. Kim Harman

**Purpose of the interview (*as described in the protocol)**

1. **Pre-study interviews** to explore a range of parent (and child/patient) views to aid the design and study procedures, to help optimize the acceptability of procedures once the trial is operational.
2. **Post-trial interviews** about participation to explore a range of parent (and child/patient) views on study participation, seeking to understand positive and negative experiences from start to finish. These will take place one participants have been randomised and participated.

**Overall structure of the interview**

1. **Introduction &** **Ethics**
2. **Topic 1:** Presenting illness & decisions to consult
3. **Topic 2:** Parent understandings about antibiotics and AMR
4. **Topic 3.1** Participation in the trial
5. **Topic 3.2** Non-participation in the trial (*for participants in the observational study)
6. **Participant demographics**
7. **Interview close**

**Interview Guide**

1. Introduction

- Hello there is that [**name of participant**]?
- Hi, my name is Catherine and I am a researcher on the ARTIC-PC study. This is a trial run by researchers are the University of Southampton which aims to improve our understanding of how to treat coughs/chest infections in children.
- I recently scheduled this interview so we could discuss your recent participation within this trial, is now still a good time to chat?
- So firstly, thank you for agreeing to take part in the trial and in the interview – myself and the team really appreciate it and I am looking forward to hearing your views of in the trial.
- The interview will take between 30 to 40 minutes, or maybe a little more depending on the kinds of things we discuss.
- Ideally, you will be talking for most of the interview, roughly an 80%/20% split between you and me. Some people find it a bit strange talking for that long but we really want to hear what you have to say as you are the expert on this topic and anything you say will be relevant and really useful for to know.
- I will be quiet at times during our talk because I’m listening to your story and also because I might be taking some notes at the same time.

Before we start the interview, just to confirm:

- That you [**took part/did not take part**] in the trial; and you agreed to be interviewed?
- That you are happy for this interview to be audio-recorded?

*Brilliant. The reason we ask that is so we have a good record of what we discussed today – if all by hand I would likely forget(!). The recording will be kept securely and it will be anonymised too so that direct quotes cannot be linked to you personally. Anonymised just means [****identifiable information removed such as names/locations***].

- You can change your mind at any point about taking part in the study even after this interview has been conducted; and you can also decline to answer any of my questions if you do not want to.
- At this point, do you have any questions for me?

Can I just confirm:

- [**Name**] of child
- [**Age**] when unwell
- Is [**name**] your first child…[**answer = Y/N**]…if **YES**…and how old are they?

**BRIDGING LINE: Okay great. Let’s start from the beginning when** [**name of child**] **first had symptoms…**

1. **Presenting illness & decisions to consult**

- Before you visited the GP, can you just talk me through what happened & when you first noticed [**name**] was not well?
  - Prompt: how long had they been like this for?
- By the time you took [**name**] to the GP, what symptoms did [**name**] have?
  - Prompt: did these symptoms appear all at the same time or did they appear separately?
- Was there anything in particular that made you take [**name**] to the GP?
  - Prompt: Have they ever had anything like this before? Can you tell me a bit more about that?
  - Prompt: Was there anything particularly concerning about these symptoms? E.g*. compared to previous illnesses/ compared to symptom severity/ other children (****if applicable****).*
- Did you have any thoughts about what might be wrong with [**name**]?
  - Prompt: Lay diagnoses (viral or infection) / read anything on the internet / Advice from [friends/ family pharmacist].
- And how were you feeling about [**name**] having these symptoms?
  - Prompt: Any particular concerns or worries?
  - Prompt: Did [**name**] being unwell have an impact on other people in the family? E.gs. *other illnesses, time off work*.
- Before visiting the GP, had you tried anything to relief [**name’s]** symptoms?
  - Prompt: over the counter medication (e.gs. *Calpol, Nurofen*, *left-over antibiotics*)?
  - Prompt: Have you used these medications before?
  - Prompt: Do you think they help?
- And what were you hoping the GP would do for **[name]** during your appointment?
  - Prompt: And what did they do? *E.g. examined, outcome [****in addition if trial****].*
  - Prompt: Were you happy with the consultation? Can you think why/why not?
- Would go to the GP again next time?
  - Prompt: Why/why not? E.gs. *Delay/ not attend/ ask particular questions*.

**BRIDGING LINE: [*Thanks for sharing that story, it was really interesting*]. I am going to move on slightly and ask you some questions about antibiotics…**

1. **Understandings about antibiotics and AMR**

- Have you ever taken antibiotics before? [**answer Y or no**]
  - Prompt: What was this for? What symptoms you were having?
  - Prompt: Do you think they were effective at treating those [**named symptoms**]?
  - Prompt: More generally, when do you think people are supposed to take antibiotics? [*So, for what kinds of symptoms?*]
  - Prompt: Do you think there are times when they should not be used? Why?
  - Prompt: Have you ever looked on the internet about abx/talked to family/HCPS? [**Answer**] What did [**they/that**] say?
- Has [**name**] or [**any of your children**] ever taken antibiotics before?
  - Prompt: For similar symptoms or something different?
  - Prompt: Do you think they helped? *Why? How do you know?*
  - Prompt: Do you have any concerns about giving antibiotics to children? E.g. *side effects*.
  - Prompt: Have you ever looked on the internet about abx and children/talked to family/HCPS?
- Have you ever heard of something called antibiotic resistance?
  - Prompt: what do you think it is?
  - Prompt: what do you think it is caused by? *E.g. linked to GPs prescribing antibiotics*?

**BRIDGING LINE: I am now going to ask you some questions about…**

**Your participation in the trial – go to page 5**

**OR**

**Your participation in the observational study – go to page 6**

1. **Participation in the trial**

- So when the GP introduced the trial, what did you initially think?
  - Prompt: *Can you remember how the GP described the trial to you? What did you think of their explanation?*
  - Prompt: Why did you decide to participate in the trial?
- Did anything in particular make you interested/ keen to participate?
  - Prompt: Did you have any concerns about taking part? (*e.g. placebo, antibiotics, time-consuming, being randomised)*?
  - Prompt: Have you ever participated in a trial before? What was it *(e.g. medically-related)? Why?*
- How did you feel about the possibility of your child taking an antibiotic?
- How did you feel about the possibility of your child taking a placebo?
  - Prompt: Had you heard of ‘placebos’ before taking part in the trial? Where?
  - Prompt: What is your understanding of a ‘placebo’?
- So the GP gave you some study materials explaining the trial, how did you find those?
  - Prompt: Easy to read? Anything we could improve upon? E*.g. length, terminology*.
  - Prompt: Did you read them before agreeing to take part in the trial? *How long take to read?*
  - Prompt: Did you understand the trial and what you would have to do before agreeing to take part?
- And during the trial you were asked to do a couple of things, how did you find:
- 1) Storing the medication? E.g. *appropriate shelf*. *Where did you store it?*
- 2) Taking the medication? E.g. *making up the solution, easy to remember, child taking it*.
- 3) Symptom diary? E.gs. *did you fill it out? How find it*?
- Overall, any positive aspects of participating in the trial? E.gs. *understanding of abx, changed views of abx*.
- Similar question, anything that you didn’t like?
- [*Asking this because we often run trials here in Southampton and if we could improve upon anything then it would be great to know so we can change it*].
- So after your GP appointment and taking this medication, when did **[name]** start to get better?
  - Prompt: Why do you think this was? E.g. time, better on own.
  - Prompt: Do you think [**name**] was given an antibiotic or a placebo? Why?
  1. Non-Participation in the trial
- So when the GP introduced the trial, what did you initially think?
  - Prompt: Can you remember how the GP described the trial to you? What did you think of their explanation?
  - Prompt: Why did you decide not to take part in the trial? [**answer = could have been GP who decided for the child**]
  - Prompt: Was there anything in particular that made you not want to take part? E.gs. *placebo, severe symptoms, antibiotic, time-consuming*.
  - Prompt: Have you ever participated in a trial before? [**answer**]…**if Y**…*What was it (e.g. medically-related)? Why?*
- You agreed to take part in the observational study? Why was that?
- So the GP gave you some study materials explaining the trial, how did you find those?
  - Prompt: Easy to read?
  - Prompt: Anything we could improve upon? E.g. length, terminology.
  - Prompt: Did you read them before agreeing to take in the observational study? How long take to read?
  - Prompt: Did you understand the study and what you would have to do before agreeing to take part?
- What about the symptom diary, how did you find that?
  - Prompt: did you fill it all out?
  - Prompt: was it helpful?
- After the consultation with the GP, when did **[name]** start to get better?
  - Prompt: Why do you think this was? E.g. *time, better on own.*
- If you were asked to participate in a trial again, do you think you would?
  - Prompt: *Why? Why not?*

1. **Demographics**

- Gender: [**name**] is a M/F?
- How old was [**name**] when s/he was unwell?
- Your relationship to [**name**]?
- Could I just take your DOB?
- How would you describe your ethnicity (e.g. White British, White Irish, Asian, Black British, etc?).
- What is your occupation?

Okay well that is the end of the questions I have for you. Is there anything else that you would like to add that we have not covered so far?

1. **Interview Close**

- Thank you so much for your time today and sharing your story with me – it was lovely to speak to you.
- Thank you for letting me record this interview - I am switching the recorder off now *(just have to find the button…).*
- Are you still happy for us to anonymise this recording and use it for our study?
- Do you have any questions for me?

Thank you so much again for taking part in the study and speaking with me today – hope you have a [*lovely day, nice weekend, good week*].
